# Human Milk Oligosaccharides and Immune System Development

**DOI:** 10.3390/nu10081038

**Published:** 2018-08-08

**Authors:** Julio Plaza-Díaz, Luis Fontana, Angel Gil

**Affiliations:** 1 Department of Biochemistry and Molecular Biology II, School of Pharmacy, University of Granada, 18071 Granada, Spain; jrplaza@ugr.es (J.P.-D.); fontana@ugr.es (L.F.); 2 Institute of Nutrition and Food Technology “José Mataix”, Biomedical Research Center, Parque Tecnológico Ciencias de la Salud, University of Granada, Armilla, 18100 Granada, Spain; 3 Instituto de Investigación Biosanitaria ibs., 18014 Granada, Spain; 4 CIBEROBN, Instituto de Salud Carlos III, 28029 Madrid, Spain

**Keywords:** human milk oligosaccharides, intestinal immune system, microbiota

## Abstract

Maternal milk contains compounds that may affect newborn immunity. Among these are a group of oligosaccharides that are synthesized in the mammary gland from lactose; these oligosaccharides have been termed human milk oligosaccharides (HMOs). The amount of HMOs present in human milk is greater than the amount of protein. In fact, HMOs are the third-most abundant solid component in maternal milk after lactose and lipids, and are thus considered to be key components. The importance of HMOs may be explained by their inhibitory effects on the adhesion of microorganisms to the intestinal mucosa, the growth of pathogens through the production of bacteriocins and organic acids, and the expression of genes that are involved in inflammation. This review begins with short descriptions of the basic structures of HMOs and the gut immune system, continues with the beneficial effects of HMOs shown in cell and animal studies, and it ends with the observational and randomized controlled trials carried out in humans to date, with particular emphasis on their effect on immune system development. HMOs seem to protect breastfed infants against microbial infections. The protective effect has been found to be exerted through cell signaling and cell-to-cell recognition events, enrichment of the protective gut microbiota, the modulation of microbial adhesion, and the invasion of the infant intestinal mucosa. In addition, infants fed formula supplemented with selected HMOs exhibit a pattern of inflammatory cytokines closer to that of exclusively breastfed infants. Unfortunately, the positive effects found in preclinical studies have not been substantiated in the few randomized, double-blinded, multicenter, controlled trials that are available, perhaps partly because these studies focus on aspects other than the immune response (e.g., growth, tolerance, and stool microbiota).

## 1. Introduction

Breastfeeding has many beneficial effects in newborns. The relative risks of diarrhea incidence, diarrhea mortality, pneumonia incidence, and pneumonia mortality are kept to a minimum in exclusively breastfed infants. These protective effects, although less robust, are also observed in partially breastfed infants when compared with milk formula-fed infants [1]. However, in addition to protecting against infection, human milk has both short-term and long-term effects, such as prevention and protection against allergic reactions; optimal behavioral, cognitive, and gastrointestinal development; and, may protect against chronic diseases, such as diabetes, obesity, hypertension, and autoimmune and cardiovascular diseases [2]. The long-term effects of human milk are related to so-called early programming [3].

Human milk contains many bioactive compounds that may affect immunity (e.g., cytokines, growth factors, hormones, digestive enzymes, transporters, and antimicrobial factors). The latter category of antimicrobial factors includes glycans, among which exists a group of oligosaccharides with different structures that are synthesized from lactose in the mammary gland. These oligosaccharides have been termed human milk oligosaccharides (HMOs). In addition, human milk contains probiotics, which reside in the microbiota of the breast tissue and may also have a role in neonate immunity [4,5].

Although HMOs were originally described and referred to as “gynolactose” by Lespagnol and Polonowski in 1930 [4], they have attracted considerable attention in recent years because of their biological roles. The HMO fraction (5–15 g/L) that is present in human milk is greater than the protein fraction (8 g/L). In fact, HMOs are the third-most abundant solid component in maternal milk after lactose (70 g/L) and lipids (40 g/L), and accordingly, they are considered to be key compounds [4,5].

HMOs have been described to inhibit (i) the adhesion of microorganisms to the intestinal mucosa; (ii) the growth of pathogens through the production of bacteriocins and organic acids; and (iii) the expression of genes involved in inflammation [4,5,6]. Although many studies regarding the composition of oligosaccharides in human milk have been published, there are few publications about the roles of these compounds in general and in immunity in particular [5,7]. The aim of this review article was to fill this gap by surveying the in vitro and in vivo effects of HMOs, focusing mainly on immunity.

## 2. Oligosaccharides in Human Milk

**Figure 1:**
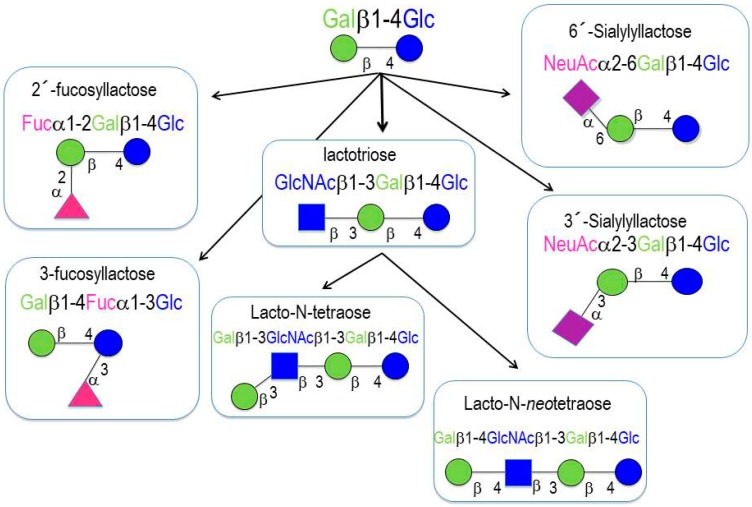
Human milk oligosaccharides (HMO) basic structures.

More than two hundred different HMOs have been identified to date. HMOs are composed of monosaccharides and monosaccharide derivatives [4]: glucose (Glc), galactose (Gal), *N*-acetylglucosamine (GlcNAc), fucose (Fuc), and sialic acid (Sia). All of the HMOs contain lactose at their reducing end, which can be elongated by the addition of β1-3- or β1-6-linked lacto-*N*-biose (Galβ1-3GlcNAc-, type 1 chain) or *N*-acetyllactosamine (Galβ1-4GlcNAc-, type 2 chain). Elongation with lacto-*N*-biose appears to terminate the chain, whereas *N*-acetyllactosamine can be further extended by the addition of either of the two disaccharides. A β1-6 linkage between two disaccharide units introduces chain branching. Branched structures are termed iso-HMOs; linear structures without branches are termed para-HMOs. Lactose or the elongated oligosaccharide chain can be fucosylated with α1-2, α1-3, or α1-4 linkages, and/or sialylated with α2-3 or α2-6 linkages. Some HMOs occur in several isomeric forms, e.g., lacto-*N*-fucopentaose or sialyllacto-*N*-tetraose [4]. HMOs with more than 15 disaccharide units have been described; such HMOs form complex structural backbones that can be further modified by the addition of Fuc or/and Sia [8,9]. HMOs are classified into three categories (Figure 1) [8,9]:

(a) Neutral (fucosylated) HMOs are neutral and contain fucose at the terminal position (e.g., 2′-fucosyllactose (2′-FL) and lactodifucopentaose). They represent 35% to 50% of the total HMO content.
(b) Neutral N-containing (nonfucosylated) HMOs are neutral, contain *N*-acetylglucosamine at the terminal position (e.g., lacto-*N*-tetraose), and represent 42% to 55% of the total HMO content.

Neutral HMOs account for more than 75% of the total HMOs in human breast milk.

(c) Acid (sialylated) HMOs are acidic and contain sialic acid at the terminal position (e.g., 2′-sialyllactose). They represent 12% to 14% of the total HMO content.

The amount and composition of HMOs vary among women. The HMO composition is determined genetically and mirrors blood group characteristics, which depend on the expression of certain glycosyltransferases. Four milk groups can be assigned based on the Secretor (Se) and Lewis (Le) blood group system, which is determined by the activity of two gene loci encoding α1-2-fucosyltransferase (FUT2, encoded by the *Se* gene) and α1-3/4-fucosyltransferase (FUT3, encoded by the *Le* gene) [6,7,8,9,10,11,12,13,14,15,16,17]. Individuals with an active *Se* locus are classified as “secretors”. The milk of secretor women is abundant in 2′-FL, lacto-*N*-fucopentaose I (LNFP I), and other α1-2-fucosylated HMOs. In contrast, non-secretor women lack a functional FUT2 enzyme, and their milk does not contain α1-2-fucosylated HMOs. Individuals with an active *Le* locus are classified as *Le*-positive. They express FUT3, which transfers Fuc with a α1-4 linkage to subterminal GlcNAc on type 1 chains. In contrast, the milk of *Le*-negative women lacks these specific α1-4-fucosylated HMOs, e.g., LNFP II [9,10,14]. Therefore, breast milk can be assigned to one of the four groups based on the expression of FUT2 and FUT3: *Le*-positive secretors (*Le*+*Se*+), *Le*-negative secretors (*Le*-*Se*+), *Le*-positive nonsecretors (*Le*+*Se*-), and *Le*-negative nonsecretors (*Le*-*Se*+) (Table 1) [9,10,14]:

This classification, however, is an oversimplification. FUT2 and FUT3 compete for some of the same substrates [19,20,21], and the levels of enzyme expression and activity translate into different profiles throughout the population. Even the milk of *Le*-negative nonsecretor women who express neither FUT2 nor FUT3 contains fucosylated HMOs, such as 3FL or LNFP III, suggesting that other *Se*- and *Le*-independent FUTs may be involved [14,22]. In addition, α1-2-fucosylated HMOs have been found in the milk of nonsecretor women near the end of lactation, and Newburg et al. suggested that FUT1 might also participate in HMO fucosylation [22]. In addition, fucosylation in preterm milk is not as well regulated as in term milk, resulting in higher within and between mother variation in women delivering preterm vs term. In fact, of particular clinical interest, the α1,2-linked fucosylated oligosaccharide 2′-fucosyllactose, which is an indicator of secretor status, is not consistently present across the lactation of several mothers that delivered preterm [23].

The amount and composition of HMOs also vary over the course of lactation. Whereas, colostrum contains as much as 20–25 g/L of HMOs, as milk production matures, HMO concentrations decline to 5–20 g/L, which still exceeds the concentration of total milk protein [4]. The milk of mothers delivering preterm infants has higher HMO concentrations than term milk [14], whereas preterm milk contains lower levels of fucosylated HMOs than term milk [24], and no differences in neutral and acidic HMOs are found between preterm and term milk [25].

## 3. The Intestinal Immune System

**Figure 2:**
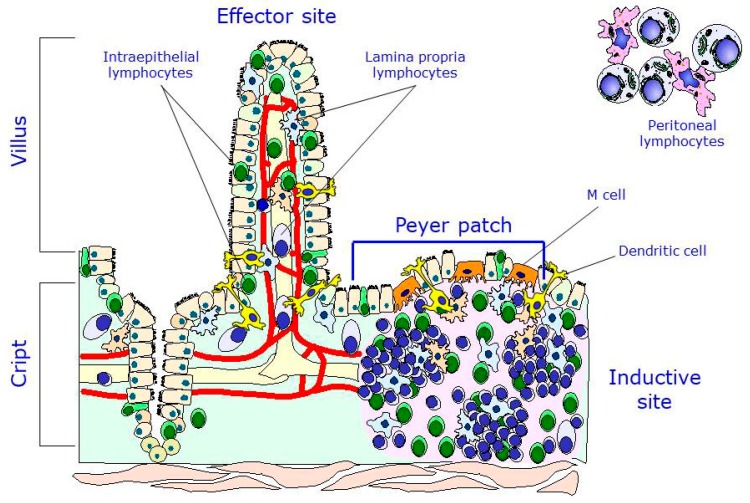
Main lymphocyte populations of the gut-associated lymphoid tissue (GALT). Modified with permission from [26].

The intestinal immune system, also known as gut-associated lymphoid tissue (GALT), is a secondary lymphoid organ that is responsible for the processing of the antigens that interact with the intestinal mucosa and of the dissemination of the immune response [26]. There are two main locations of lymphocytes in the intestine: the inductive sites, that is, where the immune response begins after stimulation by an antigen, of which the Peyer patches are the most typical, and the effector sites, that is, the ones that are responsible for executing and completing the response. There are also two main lymphocyte populations in the gut: the lymphocytes of the lamina propria (LPL), located in the internal part of the villus, and the intraepithelial lymphocytes (IELs), located among the enterocytes along the villus. It is worth mentioning that, in addition to the Peyer patch lymphocytes (PPLs), the peritoneal lymphocytes, particularly the B1 cells, are important precursors of one population of plasmatic cells found in the lamina propria. Therefore, two main inductive populations may be found at the intestinal level: the B2 cells, which reside in the Peyer patches, and the B1 cells, which reside in the peritoneum (Figure 2) [27].

The antigens that are present in the intestinal lumen are processed and transported into the Peyer patches via the M cells, which are located among the enterocytes in the epithelium. Once in the Peyer patches, the antigens interact with antigen-presenting cells (APCs), which are responsible for presenting those antigens to immature B and T lymphocytes that are residing in both the germinal centers and interfollicular regions. After their activation by antigens, the immature B and T cells drain down the lymph nodes and migrate through the thoracic duct to the bloodstream. They may circulate for a few days and later differentiate into mature effector cells that migrate to the lamina propria or memory cells, which again travel to the Peyer patches [26,27]. The so-called dendritic cells that are present in the Peyer patches and the lamina propria have been shown to form pseudopods and interact directly with antigens that are present in the intestinal lumen, after which they process the antigens and present them to other underlying cell lineages without the involvement of the M cells [28,29]. Another population of effector cells consisting of IELs may interact with antigens entering the gastrointestinal tract without following the course mentioned above. In recent years, a new type of cells, innate lymphoid cells (ILCs), have been described along with their functions [30]. ILCs are present in the intestine and other mucosae and participate in tissue homeostasis, inflammation, and autoimmunity, although their main function is the development of the gut barrier.

The potential beneficial effects of HMOs might be related to their capacity to interact with a number of receptors that are located in intestinal immune cells [31].

## 4. Beneficial Effects of HMOs

Humans lack the enzymes (sialidases, fucosidases) that break down HMOs; therefore, these compounds reach the colon intact, where they are digested by bacteria within the intestinal microbiota. In this sense, HMOs are prebiotics and they promote the growth of a favorable microbiota. Moreover, HMOs have been reported to confer additional benefits on the host, among which the three main effects are described below.

### 4.1. Inhibition of Microorganism Adhesion to the Intestinal Mucosa

The formation of the gut microbiota ecosystem is a complex but continuous process that is affected by endogenous and exogenous determinants of variability. An immediate effect at the time of birth continues for several years during childhood through subsequent stages. *Streptococcus* and *Staphylococcus* species are the most commonly identified bacterial genera in human milk, followed by *Bifidobacterium*, *Lactobacillus*, *Propionibacteria*, *Enterococcus*, and members of the *Enterobacteriaceae* family [32,33].

During early life, several external factors, such as delivery mode, feeding modality, environmental influences, antibiotic exposure, and functional food intake can affect microbiota shaping and composition [34]. The ability of the immune system to coevolve with the microbiota during perinatal life allows for the host and the microbiota to coexist in a mutually beneficial relationship [34]. Metabolic diseases are linked with disruption of both the innate and adaptive immune systems. There is evidence that some cytokines (e.g., TNF-α and IL-1) contribute to insulin resistance, thereby promoting diabetes [35] and leading to metabolic inflammation [36]. Likewise, Gram (−) lipopolysaccharide (LPS) components [37] circulate in the blood transported by LPS-binding proteins and lipoproteins, contributing to inflammation [34].

HMOs might protect breastfed infants against microbial infections due to their structural similarities to cell surface glycoconjugates utilized by microbes [38,39,40]. Experimental results have shown that oligosaccharides can provide protective effects through cell signaling and cell-to-cell recognition events, the enrichment of protective gut microbiota, and the modulation of microbial adhesion and invasion of the infant intestinal mucosa [41,42,43,44,45]. Most enteric pathogens use cell surface glycans to identify and bind to their target cells, which is the critical first step in pathogenesis.

Fucosylated HMOs have been reported to inhibit (i) the binding of several pathogens, such as *Campylobacter jejuni* [46], Norwald-like virus [47], and *Helicobacter pylori* [48], and (ii) the heat-stable enterotoxin of *Escherichia coli* [49] to intestinal cells.

The addition of HMOs was tested in T84 cell membranes to establish the inhibition of enterotoxin-producing *Escherichia coli*. The administration of HMOs repressed *E. coli* guanylate cyclase activity and cyclic GMP production in these cells [50]. Uropathogenic *E. coli* strains expressing P (Pap) and P-like (Prs) fimbriae are responsible for infections of the urinary tract. The hemagglutination that is mediated by these strains was inhibited by HMOs, especially by the sialylated fraction [51]. Fractions of HMOs were evaluated for their ability to inhibit the adhesion of *E. coli* serotype O119, *Vibrio cholerae*, and *Salmonella fyris* in differentiated Caco-2 cells. The evaluated HMOs inhibited the adhesion of these pathogens to epithelial cells [52]. Oligosaccharides from milk might block the action of PA-IIL, a fucose-binding lectin of the human pathogen *Pseudomonas aeruginosa,* through competition for the receptor and further binding [53]. In particular, a significant reduction in uropathogenic *E. coli* internalization into HMO-pretreated epithelial cells was detected without observing any binding to these cells [54]. HMOs from pooled human milk significantly reduced enteropathogenic *E. coli* strain 2348/69 (serotype O127:H6) attachment to cultured epithelial cells [55]. Likewise, treatment with HMOs reduced the invasion of human premature intestinal epithelial cells by *C. albicans* in a dose-dependent manner [56].

Colonization and invasion require the attachment of trophozoites to the host’s mucosa. HMOs reduce *E. histolytica* attachment and cytotoxicity; in fact, pooled HMOs detach *E. histolytica* by more than 80%; moreover, HMOs rescue *E. histolytica*-induced destruction of human intestinal epithelial HT-29 cells in a dose-dependent manner [57].

### 4.2. Short-Chain Fatty Acid Production by Bifidobacteria

Short-chain fatty acids (SCFAs) are an important source of energy for enterocytes and are key signaling molecules for the maintenance of gut health. HMOs can indirectly increase the production of SCFA, and these augmented levels are mediated by bifidobacteria species. SCFAs can interact with the SCFA receptors GPR41 and GPR43, increasing the intestinal secretion of polypeptide YY (PYY) and glucagon-like peptide 1 (GLP-1), respectively [58,59]. Propionate can increase free-fatty-acid uptake, possibly by affecting the lipoprotein lipase (LPL) inhibitor angiopoietin-like 4 (ANGPTL4). Acetate and propionate might also attenuate intracellular lipolysis by decreasing the phosphorylation of the hormone-sensitive lipase (HSL) through its interaction with the SCFA receptor GPR43. Propionate and butyrate could reduce the secretion of proinflammatory cytokines and chemokines, likely by reducing local macrophage infiltration [58].

Breastfed infants are typically colonized by strains of bacteria that are thought to protect, feed, and communicate with the developing intestine [60]. In 1954, Gyorgy et al. [61] conducted several studies that indicated a unique activity of HMOs as a growth factor for a *Bifidobacterium* that was isolated from the feces of an infant. Ward et al. [62,63] first demonstrated the selective growth of bifidobacteria species on intact HMOs in vitro. A number of studies have characterized the bifidobacterial moieties that specifically bind and catabolize HMOs [64,65,66,67].

*B. infantis* possesses a gene cluster that encodes transport systems and intracellular glycosyl hydrolases [41,68,69]. However, *B. bifidum* employs a different mode of catalytic activity toward HMO consumption: it exports sialidases, fucosidases, and a lacto-*N*-biosidase to release lacto-*N*-biose from HMO structures, and lacto-*N*-biose is then transported and metabolized [70]. Recently, a study revealed a multicomponent transcriptional regulation system that controls the HMO metabolism pathways in *B. breve* UCC2003 [71].

### 4.3. Inhibition of Inflammatory Genes

Although HMO-mediated changes in the infant microbiota composition or intestinal epithelial cell response may indirectly affect the infant immune system, many in vitro studies suggest that HMOs also directly modulate immune responses. HMOs may act either locally, on cells of the mucosa-associated lymphoid tissues, or at a systemic level, as 1% of HMOs are absorbed and reach the systemic circulation [4,72,73,74].

The transcriptional response of colonic epithelial cells that are treated with HMOs was investigated in HT-29 cells. The expression of several cytokines (such as IL-1β, IL-8, colony-stimulating factor 2, IL-17C and platelet factor 4 (PF4)), chemokines (such as CXCL1, CXCL3, CXCL2, CXCL6, CCL5, CCL20, and CX3CL1), and cell surface receptors (interferon γ receptor 1, IFNGR1), intercellular adhesion molecule-1 (ICAM-1), intercellular adhesion molecule-2 (ICAM-2), and IL-10 receptor a (IL10RA) in HT-29 cells was influenced by the administration of HMOs [75]. The aforementioned cytokines, chemokines, and cell surface receptors are implicated in the development and maturation of the intestinal immune response [75].

Using a cellular model with intestinal epithelial cells (T84/HCT8/FHs74) and HeLa cells, He et al. [76] investigated the effects of HMOs from colostrum in fetal human intestinal mucosa cells. These authors identified networks controlling immune cell communication, intestinal mucosal immune system differentiation, and homeostasis. HMOs treatment decreased cytokine protein levels, such as IL-8, IL-6, monocyte chemoattractant protein-1/2 and IL-1β, while increasing the levels of cytokines that are involved in tissue repair and homeostasis [76].

Gram-negative pathogenic bacteria might activate mucosal inflammation through the binding of LPS to intestinal toll-like receptor 4 (TLR4) in epithelial intestinal cells (IECs). Under in vitro conditions, IECs were treated with a strain of enterotoxigenic *E. coli* to evaluate the inhibitory effect of HMO treatment on the secretion of IL-8. Both treatments (a mixture of HMOs and 2′-FL alone) successfully decreased the LPS-dependent stimulation of IL-8 through the attenuation of CD14 induction. CD14 expression mediates the LPS-TLR4 stimulation of portions of the “macrophage migration inhibitory factors” inflammatory pathway via suppressors of cytokine signaling 2/signal transducer and the activator of transcription (STAT) 3/NF-κB [76]. The effects of different oligosaccharide fractions on leukocyte rolling and adhesion were determined. Two active compounds (3′-sialyl-lactose and 3′-sialyl-3-fucosyl-lactose) exhibit an inhibitory effect on leukocyte rolling and adhesion, decreasing the incidence of inflammatory diseases due to their anti-inflammatory activities [77].

The administration of HMOs also demonstrated growth inhibition of *Streptococcus agalactiae* (group B *Streptococcus*). This bacteriostatic activity was mediated through the action of a putative glycosyltransferase that confers resistance to oligosaccharides [78]. The human epithelial cell lines HEp-2 and HT-29 were infected with *C. jejuni* 81-176 and were treated with 2′FL to evaluate the degree of infection and inflammatory response. Treatment with 2′-FL attenuated the majority of *C. jejuni* invasion and decreased the release of IL-8 and IL-1b by 80–90% as well as decreasing the level of the neutrophil chemoattractant macrophage inflammatory protein 2 (MIP-2) [79].

Bacterial strains are not the only pathogens that are inhibited by the action of HMOs. In addition, some evidence suggests that HMOs act against viruses [6]. In particular, 2′-FL and 3-FL can structurally mimic histo-blood group antigens and block the binding of norovirus, which can cause acute gastroenteritis in humans [80]. The effects of 2′FL, 6′-sialyllactose, 3′-sialyllactose and lacto-*N*-*neo*tetraose on peripheral blood mononuclear cells (PBMCs) following respiratory viral infection were investigated in vitro. The administration of 2′-FL significantly decreased the respiratory syncytial viral load and cytokines that are associated with disease severity (IL-6, IL-8, MIP-1α) and inflammation (TNF-α, MCP-1) in airway epithelial cells. Lacto-*N*-*neo*tetraose and 6′-sialyllactose treatments decreased the influenza viral load in airway epithelial cells, and only 6′-sialyllactose decreased CXCL10 and TNF-α in respiratory syncytial virus-infected PBMCs [81]. Particularly, HMOs containing more than one unit of fucose may exhibit stronger binding capacities when compared with single fucose HMOs [82].

## 5. Immunomodulation Mediated by HMOs: Animal Studies

The immunomodulatory effects of HMOs have also been demonstrated in animal studies. The impact of HMOs on mucosal immunity and the response to rotavirus infection has been studied in piglets by Li et al. [83]. The administration of HMOs to these animals decreased the duration of diarrhea and enhanced the expression of IFN-γ and IL-10. HMO treatment failed to prevent the onset of rotavirus infection [83].

Colostrum-deprived newborn pigs were fed HMO formula (2′-FL, lacto-*N*-*neo*tetraose, 6′-sialyllactose, 3′-sialyllactose, and free sialic acid) or formula containing short-chain galactooligosaccharides and long-chain fructooligosaccharides, and were then inoculated with porcine rotavirus strain OSU at day 10. Pigs that received the HMO treatment had nearly twice as many natural killer cells and five times as many basophils as formula-fed pigs. The authors hypothesized that altered immune cell populations may mediate the effects of dietary HMOs on rotavirus infection susceptibility through the direct stimulation of immune cells, alteration of intestinal bacterial populations, modulation of intestinal barrier function, and the alteration of viral pathogenicity [84].

2′-FL is one of the most prominent short-chain oligosaccharides and it is associated with the anti-infective capacity of human milk. A murine influenza vaccination model was used to determine the effect of 2′-FL on vaccination responsiveness. 2′-FL treatment enhanced the delayed-type hypersensitivity responses and increased the serum levels of immunoglobulin (Ig) G1 and IgG2a and the expression of activation marker CD27 on splenic B cells. Finally, 2′-FL treatment had a direct effect on the maturation status and antigen-presenting capacity of bone marrow-derived dendritic cells [85]. Four-week-old male wild-type C57BL/6 mice were fed antibiotics to reduce their intestinal microbiota and then inoculated with *C. jejuni* 81–176. The effect of treatment with 2′-FL on the resulting acute transient enteric infection and immune response was evaluated. The ingestion of 2′-FL reduced *C. jejuni* colonization and the induction of inflammatory signaling molecules of the acute-phase mucosal immune response by 50–60% [79].

## 6. Effects of HMOs: Studies in Humans

The positive effects of HMOs found by in vitro and animal studies must be substantiated by findings from clinical studies. The most reliable clinical studies for assessing the benefits of HMOs are randomized, double-blinded, multicenter controlled trials (RCTs). To date, however, the number of RCTs with HMOs is very scarce, and most have focused on aspects other than the immune response.

### 6.1. Observational Studies

HMOs containing α1,2-fucosyl linkages have been shown to promote the growth of bifidobacteria. *B. longum* subsp. *infantis* and *B. bifidum* possess glycosyl hydrolase family 95 (GH95) fucosidases that act on 2′-fucosylated HMOs [68,86,87]. Mothers with FUT2 (secretor mothers) and nonsecretor mothers (with the nonfunctional enzyme) were recruited to evaluate the effects of maternal secretor status on the developing infant microbiota. Bifidobacteria colonize earlier in infants that are fed by secretor mothers than in infants fed by nonsecretor mothers. In addition, the majority of bifidobacteria were isolated from secretor-fed infants who consumed 2′-FL. Feces with high levels of bifidobacteria contained lower milk oligosaccharide levels and higher lactate levels [86]. The known HMO consumer *Bifidobacterium* were more abundant in the children of secretor mothers than those of nonsecretor mothers. The relative abundance of *Bacteroides plebeius*, a bacterium capable of utilizing sulfated polysaccharides for growth, was decreased in these children [88]. In addition, the FUT2 gene might be related to allergic disease in breastfed infants later in life. At two years of age, but not at five years, a lower incidence of IgE-associated eczema was detected in C-section-born infants who were fed breast milk containing FUT2-dependent oligosaccharides [89]. Additionally, although data suggest that higher lacto-*N*-fucopentaose III concentrations are associated with the lack of development of cow’s milk allergy, they are not required to prevent cow’s milk allergy. Therefore, other mechanisms must be in play [90].

HMOs have multiple immunomodulatory functions that influence child´s health [91]. Kuhn et al. evaluated the effects of HMO from breast milk on the survival of uninfected children born to HIV-infected mothers. Higher maternal breast milk concentrations of 2-linked fucosylated HMOs (2′-FL and lacto-*N*-fucopentaose I, as well as 3FL and lacto-*N*-fucopentaose II/III) were significantly associated with reduced mortality [91].

Breast milk samples were analyzed to determine the levels of 2-linked fucosyloligosaccharides and their relationship with the incidence of moderate-to-severe diarrhea. The incidence of diarrhea was lower in infants fed milk containing high levels of total 2-linked fucosyloligosaccharides. *Campylobacter,* and calicivirus-induced diarrhea occurred less often in infants whose mother’s milk contained high levels of 2′-FL and lacto-*N*-difucohexaose [92].

### 6.2. Randomized Controlled Trials

Marriage et al. [93] carried out a prospective, randomized, controlled, multicenter study at 28 sites throughout the United States involving 424 healthy full-term infants enrolled by day five of life to examine infant growth and the tolerance of infant formulas with a caloric density that is closer to that of human milk supplemented with HMOs and to study HMO uptake [93]. Infants were randomly divided into three formula feeding groups: (a) EF1, starter formula (64.3 kcal/100 mL) with galactooligosaccharides (GOS) (2.2 g/L) and 2′-FL at 0.2 g/L (*n* = 105); (b) EF2, starter formula (64.3 kcal/100 mL) with GOS (1.4 g/L) and 2′-FL at 1 g/L (*n* = 111); and, CF, control formula (64.3 kcal/100 mL) with only GOS (2.4 g/ L) (*n* = 101). A nonrandomized human milk-fed (HM) group of infants was also enrolled as a reference (*n* = 107). The study duration was 119 days, and the primary outcome was weight gain per day. Secondary outcomes included measures of tolerance and other anthropometric measures, formula intake, parent responses to questions that are related to satisfaction with the formula and their infant’s behavior, concentrations of 2′-FL in human milk in infant plasma and urine, and relative absorption. No significant differences were observed among any groups in growth parameters (weight, length, or head circumference) over the four-month study period. 2′-FL was present in the plasma and urine of infants fed the two formulas containing 2′-FL (EF1, EF2). No significant difference was observed in 2′-FL uptake relative to the concentration fed. All of the formulas were well tolerated and comparable in terms of average stool consistency, number of stools per day, and percentage of feedings associated with spitting up or vomiting. In conclusion, infants fed a lower caloric formula with 2′FL show growth and 2′FL uptake like breast-fed infants [93].

The same authors investigated the effects of feeding formulas supplemented with the HMO 2′-FL on biomarkers of immune function in healthy term infants [94]. For this purpose, they used a subpopulation of the infants that were enrolled in [93]. PBMCs were isolated for cellular phenotyping and stimulated ex vivo with phytohemagglutinin to examine proliferation and cell cycle progression or with respiratory syncytial virus (RSV). Cytokine concentrations were measured in plasma and in ex vivo cultures. Both groups fed EF1 and EF2 exhibited significantly different inflammatory cytokine profiles from those of the group fed the control formula (only GOS) (*p* ≤ 0.05) but not different from those of breastfed infants or from each other. The plasma concentrations of the inflammatory cytokines IL-1α, IL-1β, IL-6, and TNF-α, and the anti-inflammatory IL-1 receptor antagonist were significantly higher in the control group (only GOS) than in breastfed infants or the groups fed EF1 and EF2 (*p* ≤ 0.05). In ex vivo RSV-stimulated PBMC cultures, breastfed infants were not different from either of the groups fed the experimental formulas (EF1 and EF2), but they had lower concentrations of inflammatory cytokines TNF-α and IFN-ϒ (*p* ≤ 0.05) and tended to have lower IL-1ra, IL-6 and IL-1β than infants fed the control formula. This study suggests that infants fed a formula containing 2′FL have lower inflammatory cytokines, although being similar to those of breastfed infants [94].

Kajzer et al. [95] evaluated the gastrointestinal tolerance of infants fed infant formula supplemented with 2′-FL and short-chain fructo-oligosaccharides (scFOS) in a prospective, randomized, multicenter, double-blinded, controlled study involving 131 full-term infants enrolled between 0 and 8 days of age [95]. Infants were randomly allocated to receive either milk-based infant formula not containing oligosaccharides (*n* = 42) or milk-based infant formula containing 2′-FL (0.2 g/L) and scFOS (2 g/L) (*n* = 46). A group of 43 breastfed infants were also included. The intervention was performed for 35 days. The primary outcome was the average mean rank stool consistency (MRSC) from study day 1 to visit 3, calculated from stool records. From study day 1 to visit 3, no difference in stool consistency was observed. At visit 3, there were no differences between groups in the average volume of study formula intake, the number of study formula feedings per day, anthropometric data, or percentage of feedings with spitting up or vomiting. The conclusion of this study was that the formula containing 2′FL was well tolerated in infants as evidenced by stool consistency, formula intake, anthropometric data and percent feedings with spitup/vomit similar to that of infants fed a formula without oligosaccharides or breast milk [95].

The effects of feeding infant formulas supplemented with two human milk-identical oligosaccharides, 2′FL, and lacto-*N*-*neo*tetraose (LNnT), on infant growth, tolerability, gut microbiota, and medication use were investigated in a randomized, controlled, multicenter trial in Italy and Belgium [96,97,98]. One hundred and seventy-five healthy, full-term infants were randomly allocated after birth (between 0–14 days of age) to one of the two formula feeding groups: intact cow’s milk-based whey-predominant infant formula with the addition of 2′-FL (1.0 g/L) and LNnT (0.5 g/L) (test group, *n* = 88) or intact cow’s milk-based whey-predominant infant formula (control group, *n* = 87). The formulas were given up to six months of age (exclusive formula feeding up to four months). A group of 38 exclusively breastfed infants were enrolled at three months as a reference (breastfed group). The primary endpoint was weight gain through four months of age. The secondary endpoints were anthropometry, stool characteristics, stool microbiota (at 3 and 12 months of age, obtained using 16S rRNA gene sequencing and metagenomics), stool metabolic signature (at 3 and 12 months of age, obtained using proton NMR-based metabolic profiling,), digestive tolerance, and morbidity (reported by parents) through 12 months of age. The weight gain up to four months of age of infants fed formula supplemented with 2′-FL and LNnT was not inferior to the weight gain of infants fed unsupplemented formula. The mean weight, length, head circumference, and BMI up to 12 months of age of infants fed formulas with or without 2′-FL and LNnT were close to the WHO Growth Standards and did not differ between the two groups. Digestive tolerance was similar between the two groups. Infants receiving formula containing 2′-FL and LNnT had significantly fewer parental reports of lower respiratory tract infections (19.3% vs. 34.5%; OR 0.45, 95% CI 0.21–0.95; *p* = 0.027), particularly bronchitis (10.2% vs. 27.6%; OR 0.30, 95% CI 0.11–0.73; *p* = 0.004), up to 12 months of age (42% vs. 60.9%; OR 0.47, 95% CI 0.24–0.89; *p* = 0.016), and lower medication (antibiotics up to 12 months of age, antipyretics (15.9% vs. 29.9%; OR 0.44; 95% CI 0.2–0.98; *p* = 0.032) up to four months of age) than infants fed formula without 2′FL and LNnT [96]. In conclusion, feeding infant formula containing two HMOs (2′FL and LNnT) during the first six months of age are safe, well-tolerated, and supports age-appropriate growth [81]. Also, the observed effects on reduced morbidity and medication use in infants up to 12 months of age, when feeding formula with HMOs, suggest that 2′FL and LNnT may provide immune benefits [81].

In a second work, these authors reported the effects of 2′-FL and LNnT on the infant gut microbiota [97]. The microbiota composition of infants that were fed formula containing 2′-FL and LNnT was significantly different from that of infants fed nonsupplemented formula (*p* < 0.001) at the genus level and closer to that of breastfed infants at three months of age. Three main bacterial genera (*Bifidobacterium, Escherichia,* and *Peptostreptococcaceae*) showed significant differences in infants fed formula with or without 2 ‘FL and LNnT at three months of age: greater abundance of beneficial *Bifidobacterium* (*p* < 0.01); and, lower abundance of potentially pathogenic *Escherichia* (*p* < 0.01) as well as of unclassified *Peptostreptococcaceae* (*p* < 0.05) were observed in infants fed formula containing 2′-FL and LNnT as compared to infants fed nonsupplemented formula. These values were closer to the levels that were observed in breastfed infants. The biochemical composition of the stools was explored by the quantitative profiling of major metabolites to gain additional information on the compositional aspects. The stool contents of some amino acids (phenylalanine, tyrosine, isoleucine), some SCFAs (propionate, butyrate), and some organic acids (lactate) in infants fed formula with 2′-FL and LNnT tended to be closer to those that were observed in breastfed infants than those in infants fed nonsupplemented formula [97]. This study shows that a formula supplemented with 2′FL and LNnT shifts stool microbiota and metabolic signatures of infants born at term closer to that of breastfed infants [97].

Finally, in a third work, the same authors found that, at three months, the microbiota composition in the test group appeared closer to that of the breastfed group than to that of the control group according to alpha (within group) and beta (between groups) diversity analyses of the microbiota and the distribution of microbiota community types (A, B, and C). Supplementation with both HMOs decreased the number of infants with formula-specific C-community (fecal community type/FCT C) and increased those with the breastfed-specific B-community (FCT B). Cumulative antibiotic use up to 12 months was associated with the FCT distribution at three months. Infants with FCT B at 3 months were less likely to be treated with antibiotics (OR 0.4 (95% CI, 0.17–0.93; *p* = 0.033)), while infants with FCT C were more likely to be treated with antibiotics during the first 12 months (OR 3.3 (95% CI, 1.54–7.02; *p* = 0.0025)). The microbiota community type at three months was not associated with other parent-reported infection-related morbidities [98]. This study confirms the microbiota results of the previous one [97] and shows that infants with a breastfed specific microbiota community type (FCT B) are less likely to need antibiotics.

## 7. Conclusions and Future Perspectives

HMOs have been described to exert immunomodulatory effects. Many in vitro studies suggest that HMOs directly modulate immune responses, acting either locally on cells of the mucosa-associated lymphoid tissues or systemically to inhibit the expression of inflammatory genes, mainly cytokines. Studies in animals have shown that the administration of HMOs decreases the duration of diarrhea and enhances the expression of cytokines. Observational studies in humans have documented that certain HMOs promote the growth of bifidobacteria, which in turn affect the production of lactate and SCFAs that mediate systemic effects, including immunomodulation. Likewise, the intake of 2-linked fucosylated HMOs is associated with a reduced incidence of eczema, as well as reduced mortality in children whose mothers were infected with HIV. Moreover, HMOs play a protective role against bacterial and viral acute diarrhea. HMOs seem to protect breastfed infants against microbial infections due to their structural similarities to pathogen cell surface molecular patterns, and the protective effect has been found to be exerted through cell signaling and cell-to-cell recognition events, enrichment of the protective gut microbiota, and the modulation of microbial adhesion and invasion of the infant intestinal mucosa. Finally, infants fed formula supplemented with selected HMOs exhibit a pattern of inflammatory cytokines that is closer to that of exclusively breastfed infants.

Although new analytical systems that take advantage of the separation of complex carbohydrates by HPLC and identification by mass spectrometry have been developed in recent years, there is a need for a simpler standardized methodology to estimate the HMO patterns and contents in human milk worldwide.

Concerning the potential mechanisms of action of HMOs on immunity, new experimental approaches using human cell intestinal lines and animal models are necessary. In particular, there is a need to know how HMOs, either directly or indirectly though signaling cascade interactions, affect the expression of genes that are involved in antigen tolerance, the development of the GALT, and the response to pathogens, both bacteria and viruses.

A recent development in infant formulas is the incorporation of selected HMOs, mainly neutral, due to new biotechnological processes that are can incorporate human mammary gland glycosyltransferases. However, these formulas lack acidic HMOs, which are known to play relevant biological roles in the inhibition of pathogen and toxin adhesion to the intestine.

The number of RCTs that have evaluated the influence of HMOs on infant health is very scarce, and the number of subjects was calculated to determine the safety and tolerance of the HMO-supplemented formulas but not to generate actual evidence of the potential preventive effects of HMOs against infectious diseases. Therefore, new RCT studies in infants with the appropriate power should be designed to ascertain the roles of HMOs in the prevention of diarrhea, pneumonia, and other respiratory diseases.

## Figures and Tables

**Table 1:** Human milk oligosaccharide (HMO) groups.

Genes	*Lewis*+	*Lewis*-
*Secretor*+	*Se*+*Le*+	*Se*+*Le*-
	Able to secrete all HMOs	Able to secrete 2´FL, 3FL, LNFP-I, LNFP-III
*Secretor*-	*Se*-*Le*+	*Se*-*Le*-
	Able to secrete 3FL, LNFP-II, LNFP-III	Able to secrete 3FL, LNFP-III, LNFP-V

Abbreviations: FL, fucosyllactose; LNFP, lacto-*N*-fucopentaose. Taken from [18].
